# Isolation of levoglucosan-utilizing thermophilic bacteria

**DOI:** 10.1038/s41598-018-22496-2

**Published:** 2018-03-06

**Authors:** Shintaro Iwazaki, Hirokazu Hirai, Norihisa Hamaguchi, Nobuyuki Yoshida

**Affiliations:** 1 Department of Engineering, Graduate School of Integrated Science and Technology, Shizuoka University, 3-5-1 Johoku, Naka-ku, Hamamatsu, 432-8561 Japan; 2 Nihon Shokuhin Kako Co., Ltd, 30 Tajima, Fuji, 417-8530 Japan

## Abstract

We previously developed an industrial production process for novel water-soluble indigestible polysaccharides (resistant glucan mixture, RGM). During the process, an anhydrosugar—levoglucosan —is formed as a by-product and needs to be removed to manufacture a complete non-calorie product. Here, we attempted to isolate thermophilic bacteria that utilize levoglucosan as a sole carbon source, to establish a removing process for levoglucosan at higher temperature. Approximately 800 natural samples were used to isolate levoglucosan-utilizing microorganisms. Interestingly, levoglucosan-utilizing microorganisms—most of which were filamentous fungi or yeasts—could be isolated from almost all samples at 25°C. We isolated three thermophilic bacteria that grew well on levoglucosan medium at 60°C. Two of them and the other were identified as *Bacillus smithii* and *Parageobacillus thermoglucosidasius*, respectively, by 16S rDNA sequence analysis. Using *B*. *smithii* S-2701M, which showed best growth on levoglucosan, glucose and levoglucosan in 5% (wt/vol) RGM were completely diminished at 50°C for 144 h. These bacteria are known to have a biotechnological potential, given that they can ferment a range of carbon sources. This is the first report in the utilization of levoglucosan by these thermophiles, suggesting that our results expand their biotechnological potential for the unutilized carbon resources.

## Introduction

Dietary fibers—polysaccharides that are recalcitrant to hydrolyzing enzymes in the human living body^1,2^—provide numerous health benefits and include cellulose, hemicellulose, pectin, chitin, and chitosan. However, these polysaccharides are insoluble in water and water-soluble dietary fibers would be useful foods and beverage additives. The efficacy of water-soluble dietary fiber has been proved as improving serum liquid values^3,4^, increasing carbohydrate metabolism^5^ and intestinal flora^6^, and modulating immune functions^7,8^.

Recently, we have developed a novel procedure to synthesize water-soluble indigestible polysaccharides (resistant glucan mixture, RGM) by heating glucose at 180°C in the presence of activated carbon^9^. The RGM has a highly branched structure with α- and β-1,2-; 1,3-; 1,4-; 1,6-linkages of glucose, which may confer indigestibility and water solubility. This method is simple and efficient, and the properties of RGM as a food additive are superior to those of other commercial dietary fibers. In the industrial process for producing RGM, the first polycondensation of glucose with activated carbon at 180°C produces approximately 10% (wt/vol) of mono- and disaccharides as by-products. The monosaccharide fraction contains considerable amounts of glucose and an anhydrosugar, levoglucosan (1,6-anhydro-β-d-glucose, LG). LG is currently attracting worldwide attention, because anhydrosugars such as LG, cellobiosan, mannosan, and galactosan are known to be produced from the burning of biomass and used as indicators of biomass burning^10,11^. Based on the report that 24 mg of anhydrosugars is present per g of organic carbon in wildfire smoke^12^, Lian *et al*. estimated that 90 million metric tons of anhydrosugars are produced every year^13^. The most numerous sugar on our planet is glucose and the largest amount of LG is expected among anhydrosugars. These estimations suggest that LG-utilizing microorganisms are widely distributed in nature. Approximately twenty years ago, Yasui *et al*. attempted to isolate LG-utilizing microorganisms under ambient temperature conditions^14^. They isolated basidiomycetes, red yeasts such as *Sporobolomyces salmonicolor* and *Cryptococcus albidus*, and filamentous fungi belonging to the genera of *Aspergillus*, *Penicillium*, and *Rhizopus*. They suggested that ATP-dependent LG kinase was involved in the metabolism of LG in the eukaryotic microorganisms. We also isolated red yeasts and filamentous fungi in this study. Yasui *et al*. also found LG metabolism in a mesophilic bacterium, *Arthrobacter* sp. I-522, which may involve a NAD-dependent LG dehydrogenase^15,16^. However, no further examination has been performed in these LG-utilizing microorganisms.

It was reported that levoglucosan instilled and/or exposed to mice and human was excreted to their urine, suggesting that LG is not metabolized in living bodies^17,18^. However, mono- and disaccharide fractions containing not only glucose but also anhydrosugars described above should be removed to manufacture a higher grade of RGM as a commercial dietary fiber. Therefore, in this study, we attempted to isolate thermophilic bacteria that utilize LG as a sole carbon source, to develop a biological removing process for these monosaccharides. Given that the first step of the RGM production is heating process at 180°C, a removing process at higher temperatures would be more effective in reducing cooling costs and contamination of other microorganisms. Consequently, we isolated three LG-utilizing thermophilic bacteria and established an LG-removing process using the isolates. To our knowledge, this is the first reports on the utilization of LG by thermophilic bacteria.

## Results

### Isolation of LG-utilizing thermophiles

The synthetic medium containing LG as the sole carbon and energy source was used for the isolation at 25°C and 60°C. Interestingly, microbial growth was observed in culture media inoculated with almost all natural samples (838 samples of soil, plant leaves, pond water, etc.) at 25°C. Several microorganisms were isolated from the culture media that showed high microbial growth, using LG-agar plates. All of the isolates seemed to be filamentous fungi and yeasts, based on colony and microscopic observations. At 60°C, approximately one-sixth of the natural samples showed microbial growth and 30 bacteria were isolated from the culture media. Among them, three that grew well on LG at 60°C were used for further examination. The soil samples from which the three bacteria were isolated had been collected from the Shizuoka and Nagano prefectures in Japan under normal ambient conditions.

### Characterization of isolated thermophiles

**Figure 1:**
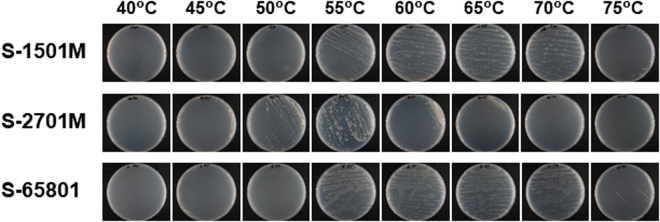
Growth of the isolated thermophiles at various temperatures. The isolates, S-1501M, S-2701M, and S-65801 were streaked onto LG-agar plates and incubated for 30 h at 40–75°C.

**Figure 2:**
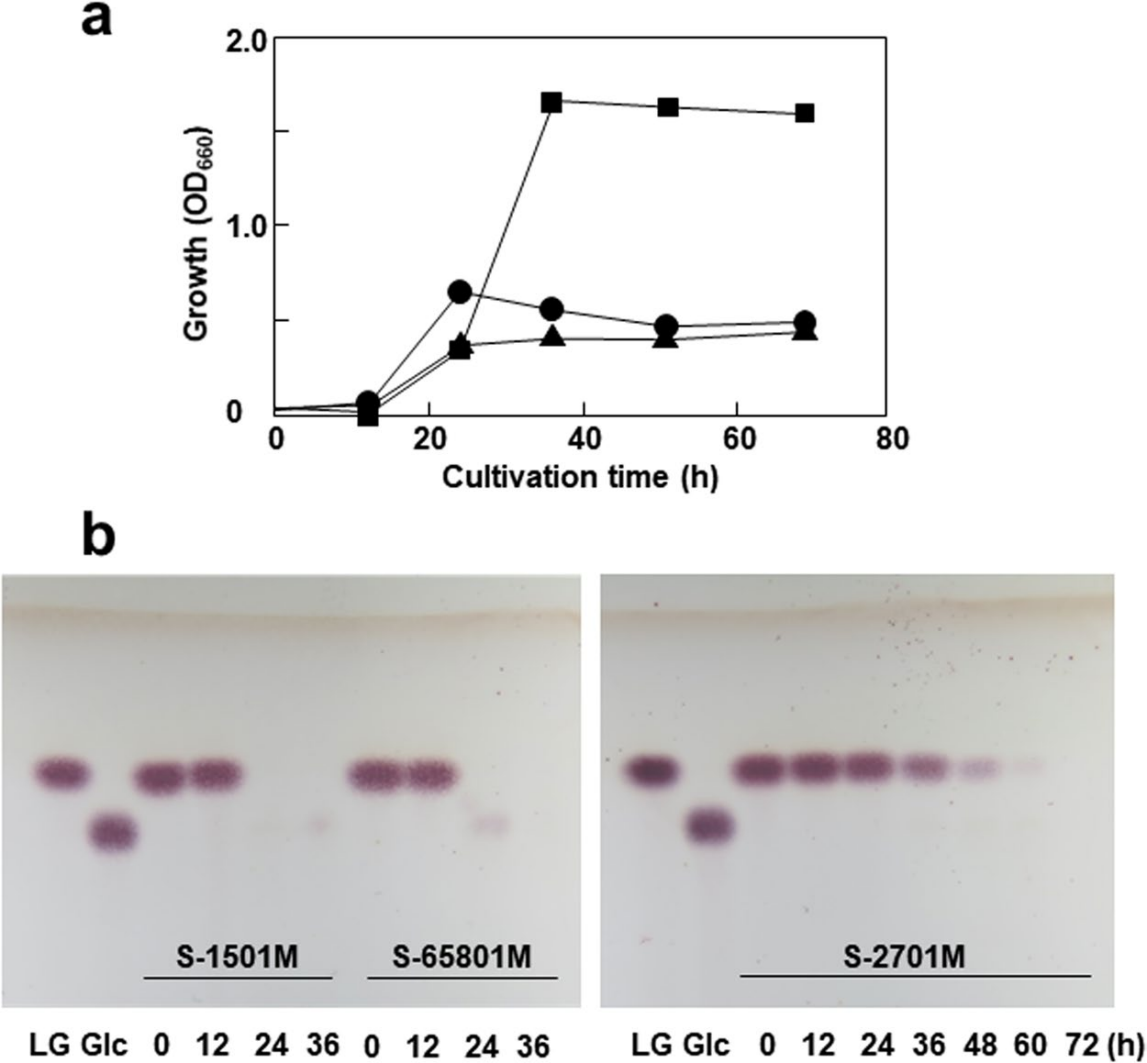
Growth and LG utilization of the isolate thermophiles in LG medium. (**a**) Each isolate was inoculated into 50 ml of LG medium as the OD_660_ of the medium was 0.02. The cultivation was carried out at 50°C for S-2701M and 60°C for S-1501M and S-65801 with reciprocal shaking at 100 rpm. Circle, square, and triangle symbols represent the growth of S-1501M, S-2701M, and S-65801, respectively. (**b**) Culture medium was withdrawn at the time indicated in the figure and centrifuged. Ten microliters of the supernatant was spotted on to a silica gel plate and developed with a solvent system composed of *n*-butanol/acetic acid/water (4:5:1). The spots were detected by anisaldehyde solution. One milligram per milliliter of glucose and LG were used as standards.

The isolated bacteria grew well at 50–70°C and the growth decreased below 50°C and above 70°C, indicating that the bacteria were thermophiles (Fig. 1). Optimum growth temperatures for S-1501M, S-2701M, and S-65801 were 60–65, 50–55, and 55–65°C for further examinations, respectively. 16 S rDNA sequence analysis revealed that S-1501M and S-2701M were identified as *Bacillus smithii* with each identity of 99.9%, whereas S-65801 was identified as *Parageobacillus thermoglucosidasius* with identities of 99.0%. Each thermophile was cultivated in a liquid medium containing LG as the sole carbon source at each optimum temperature and decrease of LG in the medium was examined by TLC analysis. As shown in Fig. 2a, S-2701M showed the best growth among the isolates, whereas the growth of all isolates reached a plateau after 1 day. Examinations of culture conditions, in which alternative nitrogen sources such as (NH_4_)_2_SO_4_ and NaNO_3_ were used, and pH decrease was prevented by adding potassium phosphate buffer, could not improve the growth of these thermophiles. TLC analysis (Fig. 2b) showed that LG in the media completely disappeared after 1 day for S-1501M and S-65801, which corresponded to the respective growth plateaus. In S-2701M, LG utilization continued during the stationary phase, while the final growth was higher than those of the other strains.

Since *B*. *smithii* is known to be able to utilize a range of carbohydrates that can be derived from lignocellulosic feedstocks^19^, utilization of carbohydrates was examined for the thermophiles isolated in this study and a *B*. *smithii* type strain DSM 4216, the genome of which has been completely sequenced. As shown in Table 1, the *B*. *smithii* type strain could not utilize anhydrosugars such as LG and cellobiosan, whereas S-2701M and S-65801 utilized both anhydrosugar. S-1501M showed similar utilization as the type strain and did not use cellobiosan as the carbon source. S-65801 could utilize a variety of the carbohydrates including mono-, di-, and polysaccharides.

**Table 1:** Carbohydrate utilization of the isolated thermophiles and *B*. *smithii* type strain.

Carbohydrate	S-1501M	S-2701M	S-65801	*B*. *smithii* type strain
Glycerol	++	+	+	+
Erythritol	++	−	−	+
d-Arabinose	++	−	−	+
l-Arabinose	++	−	++	++
d-Ribose	++	+	++	+
d-Xylose	++	−	++	++
l-Xylose	+	−	−	−
Methyl-β-d-xylopyranoside	+	−	−	−
d-Galactose	+	−	+	−
d-Glucose	++	++	++	++
d-Fructose	++	++	++	++
d-Mannose	+	−	++	−
l-Rhamnose	−	−	+	−
d-Mannitol	++	+	++	+
d-Sorbitol	+	−	++	−
*N*-Acetylglucosamine	−	−	++	−
Salicin	−	−	+	−
d-Cellobiose	−	−	++	−
d-Maltose	−	+	++	−
d-Melibiose	−	−	+	−
Sucrose	−	−	++	−
Trehalose	++	++	++	++
Starch	−	−	++	−
Glycogen	−	−	++	−
d-Turanose	−	−	+	−
LG	++	++	++	−
Cellobiosan	−	++	++	−

If bacterium utilizes carbohydrate in each medium provided in the kit, the color of the medium turns yellow from red. ++, red color; +, orange to yellowish red; −, yellow. None of bacteria tested showed utilization of the following carbohydrates provided in the kit: d-Adonitol, l-sorbose, dulcitol, inositol, methyl-α-d-mannopyranoside, methyl-α-d-glucopyranoside, amygdalin, arbutin, esculin ferric citrate, d-lactose, inulin, d-melezitose, d-raffinose, xylitol, gentiobiose, d-lyxose, d-tagatose, d-fucose, l-fucose, d-arabitol, l-arabitol, d-gluconate, 2-keto-d-gluconate, and 5-keto-d-gluconate.

### Removal of monosaccharide fractions in RGM by the isolated thermophiles

**Figure 3:**
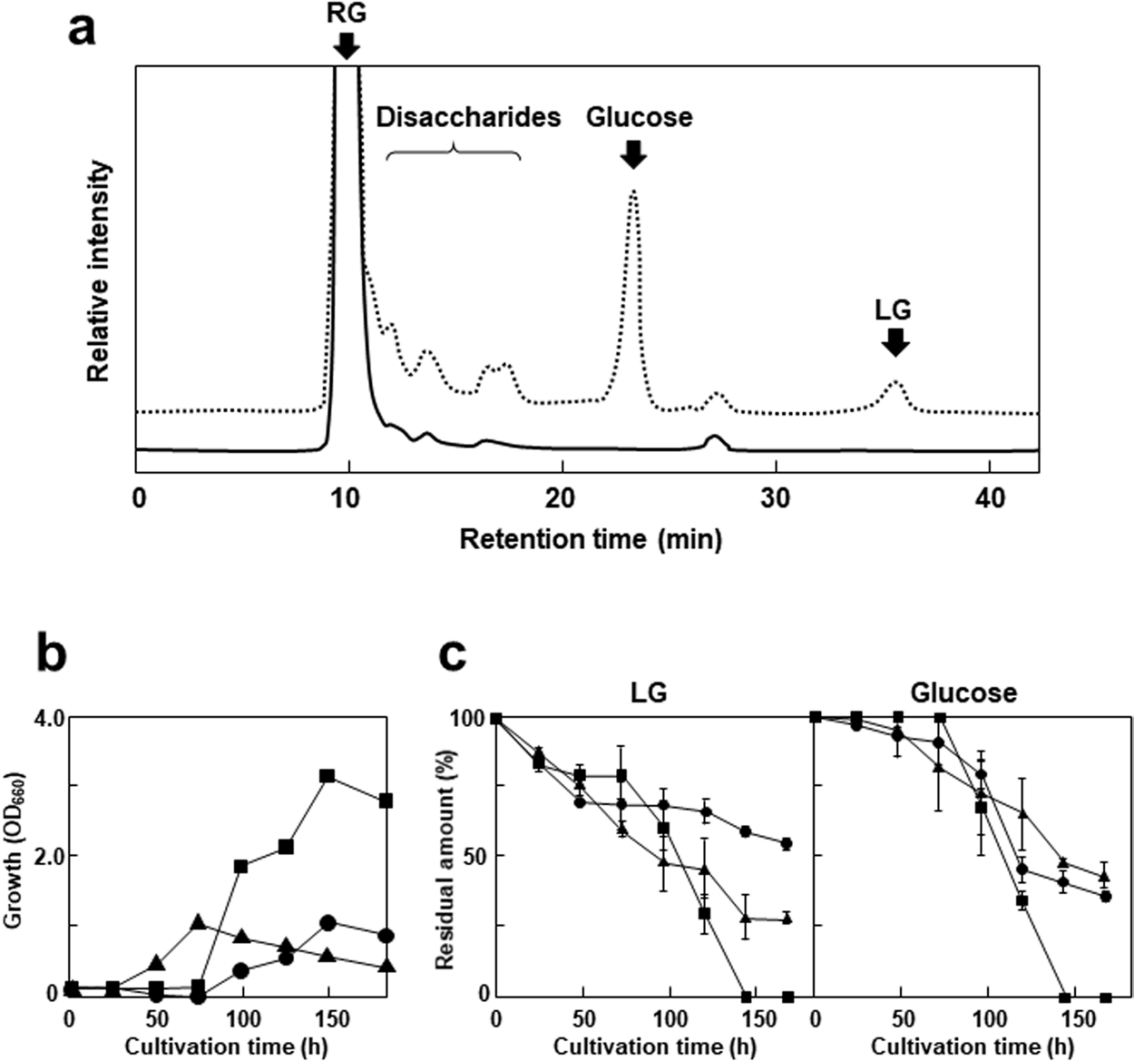
Bacterial treatment of RGM. The isolated thermophiles were cultivated in 5% (wt/vol) RGM medium at 50°C for S-2701M and 60°C for S-1501M and S-65801 with reciprocal shaking at 100 rpm. The culture filtrates were subjected to HPLC assay to evaluate the degradation of sugar fractions. (**a**) HPLC chromatogram of the culture filtrate of S-2701M after 144-h cultivation. Dotted and solid lines represent the chromatograms before and after treatments, respectively. (**b**) Growth of the isolated in the medium. (**c**) Glucose and LG consumption rates in RGM by the isolates. Circle, square, and triangle symbols represent the growth of S-1501M, S-2701M, and S-65801, respectively. The standard deviations of results from three independent experiments are displayed by error bars.

Each isolated thermophile was cultivated in a medium containing 5% (wt/vol) RGM at at the optimum temperature to remove the monosaccharide fractions. Figure 3a shows that the peaks corresponding to glucose and LG disappeared completely after 144-h cultivation of S-2701M. Furthermore, fractions that may contain disaccharides also decreased and consequently, the purity of RGM increased. These results suggested that S-2701M can be used for removal of unnecessary fractions in RGM without loss of the properties of RGM as a dietary fiber. S-2701M grew better than the other strains RGM medium like that in LG medium (Fig. 3b) and the other two strains could not completely utilize the monosaccharide fractions in RGM (Fig. 3c).

### Enzyme activity involved in the metabolism of LG in the isolates

**Figure 4:**
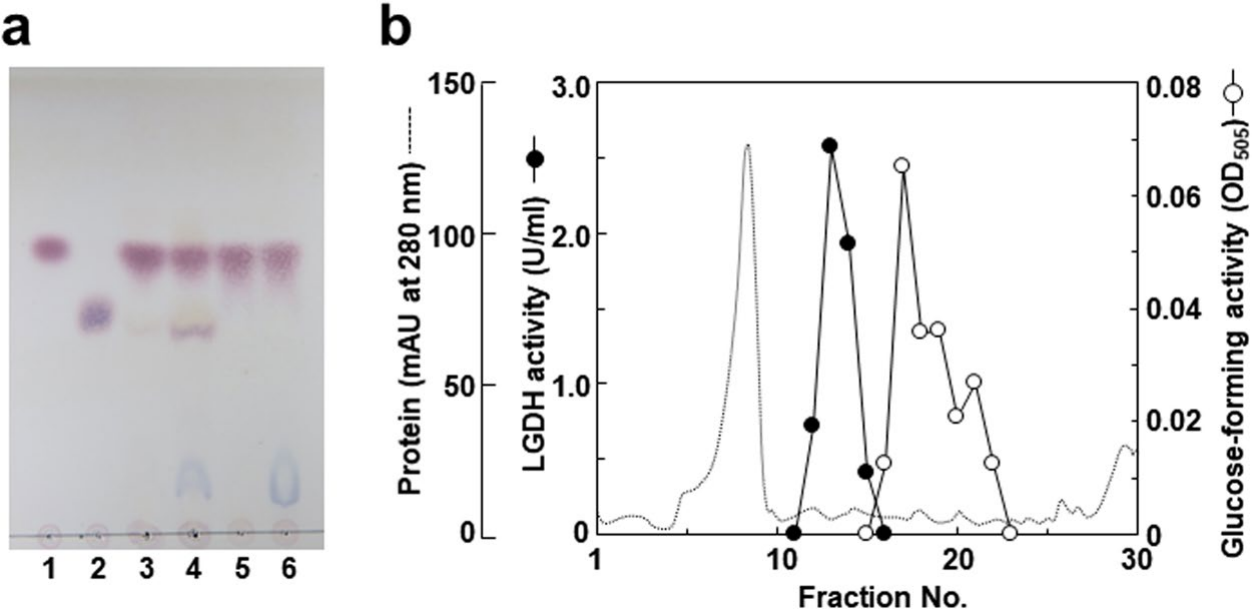
TLC analysis and anion-exchange chromatography of the enzyme activities against LG. (**a**) LG (1 mg/ml) was incubated at 50°C for 5 h in 60 mM Tris-HCl (pH 8.0) containing enzyme and/or NAD, and 10 μl of the reaction mixture was spotted on to a silica gel plate. TLC analysis was performed under the same conditions described in the legend of Fig. 2. Lane 1: LG (1 mg/ml), lane 2: glucose (1 mg/ml), lane 3: LG + crude extract (50 μg/ml), lane 4: LG + crude extract (50 μg/ml) + NAD (2 mM), lane 5: LG + LGDH (0.2 μl/μl of No. 13 fraction in Fig. 4b), lane 6: LG + LGDH (0.2 μl/μl) + NAD (2 mM). (b) Crude extract obtained from 30 ml of LG medium was applied to a HiTrap Q column (1 ml) equilibrated with 50 mM Tris-HCl buffer (pH 8.0). The column was washed with 5 ml of the same buffer and eluted with a linear gradient of 0–0.3 M KCl (1 ml per fraction). For glucose-forming activity, 1 mg/ml of LG, 10 μl of each fraction, 10 μl of No. 13 fraction, and 2 mM NAD were incubated at 40°C for 5 h in 30 mM Tris-HCl buffer (pH 8.0), and glucose in the reaction mixture was assayed by glucose oxidase method.

Since S-2701M showed best growth in LG medium and highest consumption of LG and glucose in RG medium, the enzyme reaction for LG was examined in crude extract of S-2701M. When the crude extract was incubated with LG, a slight spot corresponding to glucose was observed in TLC analysis (Fig. 4a, lane 3). Addition of NAD in the reaction mixture enhanced the glucose formation (Fig. 4a, lane 4). Until now, two enzymes have been reported in LG-utilizing microorganisms. One of them is LG kinase, which catalyzes ATP-dependent phosphorylation of LG to produce glucose 6-phosphate, and is found in fungi and yeasts^14^. The other is NAD-dependent LG dehydrogenase found in *Arthrobacter* sp.^15^. Crude extracts of the isolated thermophiles had a remarkable NAD-dependent LG dehydrogenase (LGDH) activities (20.6, 43.2, and 25.3 mU/mg for S-1501M, S-2701M, and S-65801, respectively), whereas LG kinase activity was not detected. The LGDH activities were highly specific for NAD as the coenzyme, and none of activities were detected with NADP or without NAD. In anion-exchange chromatography for the crude enzyme (Fig. 4b), single peak for LGDH was observed, whereas none of glucose-forming activity was found using LG as the substrate in each fraction. However, when No. 13 fraction, which showed the highest LGDH activity, was added to the reaction mixture, a peak for glucose-forming activity was observed separately from that for LGDH. TLC analysis also showed glucose was not detected from LG with the No. 13 fraction (Fig. 4a, lanes 5, 6). These results suggested that the same metabolism of LG as that in *Arthrobacter* sp. is involved in LG utilization by the thermophiles.

## Discussion

**Figure 5:**
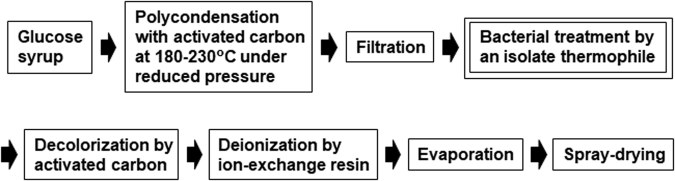
Novel industrial process for RGM production.

We previously tried to introduce a simulated moving bed chromatography to remove the mono- and disaccharide fractions in RGM^9^. This process used eight identical columns with a cation-exchange resin and produced a large amount of waste containing saccharides. The thermophilic bacteria isolated in this study can be used to remove not only LG but also glucose in RGM. By introduction of biological process using the isolates, these chromatographic separation can be removed and only ion-exchange treatment can provide purified RGM preparation (Fig. 5). Actually, we have tried pilot examination with the biological process and confirmed the physical and chemical properties and the taste of the final product was almost the same as those of RGM produced by the current process. Further examinations, however, are needed to apply the results in this study to practical industrial production processes of RGM. For example, construction of a bioreactor containing high density of one of the isolates may be effective without monosaccharide waste (Fig. 4). Furthermore, the novel LG-removing process established in this study could also be applied for industrial production processes for other carbohydrate-related products. Actually, it is known that LG is contained not only in RGM but also in other commercial dietary fibers, such as indigestible dextrin and polydextrose, the production processes of which contain a heating process.

While all of the LG-utilizing microorganisms reported so far are mesophiles, in this study, we isolated three thermophiles identified as *B*. *smithii* and *P*. *thermoglucosidasius* that can utilize LG at 50–70°C, which are more suitable for removal of LG and glucose at a higher temperature in current industrial process. In this study, we also attempted to isolate LG-utilizing microorganisms under ambient conditions and isolated a lot of red yeasts. All of the red yeasts had the LG kinase activities (data not shown), which was consistent with the previous report^14^. These red yeasts grew well in a liquid medium containing LG as a sole carbon source, but they did not grow at over 50°C. The thermophiles isolated in this study are known to have a biotechnological potential, because they are able to ferment a variety of carbon sources, such as d-glucose, d-xylose, l-xylose, l-arabinose, d-fructose, l-sorbose, d-tagatose, and l-rhamnose^20^ and produce lactate and other green building block chemicals^21,22^. As described above, the substantial amount of anhydrosugars such as LG is expected to be in nature, microorganisms that utilize and convert anhydrosugars into other chemicals such as biofuel would be useful for processing unused biomass. In this study, it was revealed that *B*. *smithii* type strain DSM 4216 could not utilize anhydrosugars such as LG and cellobiosan. This is the first report on the utilization of levoglucosan by these useful thermophiles, suggesting that our results could expand their biotechnological potential for the unutilized carbon resource. S-2701M is expected to be an appropriate strain for LG removal in the RGM production process, but S-65801 may be more useful as for the effective use of lignocellulosic biomass.

**Figure 6:**
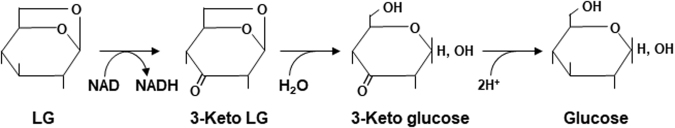
Postulated LG metabolism in S-2701M.

While the crude extract of S-2701M had an apparent glucose-forming activity from LG, the activity diminished in anion-exchange chromatography. The crude extract also had the remarkable NAD-dependent LGDH activity and the glucose-forming activity was restored when LGDH was added to the assay in the anion-exchange chromatography. However, this glucose-forming activity was not detected again in subsequent gel permeation chromatography (data not shown), suggesting that at least three enzymes is involved in the formation of glucose from LG in S-2701M. These results is well-consistent with those in the LG metabolism in *Arthrobacter* sp.^15^ and it is suggested that S-2701M can convert LG to glucose in three steps, in which LGDH catalyzes the initial reaction, prior to glycolytic pathway (Fig. 6). It is noteworthy that the enzymes involved in conversion of LG to glucose may not be affected by catabolite repression, because LG was utilized firster than glucose in RGM (Fig. 3c). Given that S-2701M was identified as *B*. *smithii* and the type strain did not utilized LG, the comparative genomics can elucidate the genes encoding the enzymes catalyzing LG to glucose.

## Materials and Methods

### Chemicals

RGM was synthesized from glucose syrup with activated carbon as described previously^9^. RGM was further treated with α-amylase and glucoamylase^9^ and used in this study. LG was prepared from RGM by fractionation with a cation-exchange resin^9^. Cellobiosan was purchased from Santa Cruz Biotechnology, Inc (TX, USA).

### Screening of LG-utilizing microorganisms

Basal medium used throughout this study was composed of 1.0 g of NH_4_Cl, 1.0 g of K_2_HPO_4_, 1.0 g of KH_2_PO_4_, 0.5 g of MgSO_4_ · 7H_2_O, 0.1 g of CaCl_2_ · 9H_2_O, 0.1% (vol/vol) Trace Metal Mix A5 with Co (Merck Ltd., Tokyo, Japan) in 1,000 ml of deionized water. The vitamin mixture had the following composition: 1 mg of thiamine-HCl, 2 mg of riboflavin, 2 mg of Ca-pantothenate, 2 mg of pyridoxine-HCl, 0.1 mg of biotin, 1 mg of *p*-aminobenzoic acid, 2 mg of nicotinic acid, and 0.1 mg of folic acid in 100 ml of deionized water. Purified agar (Nacalai Tesque, Kyoto, Japan) was used to prepare agar plates. For isolation of LG-utilizing microorganisms, a small amount of each natural sample, which was collected from various environments was added to 3.0 ml of the basal medium containing 1% (wt/vol) LG. Two types of culture medim (pH 5.5 and 7.0) were used for each natural sample. The cultivation was carried out at 25 or 60°C with a reciprocal shaking. If turbidity of the culture was observed, the culture was streaked onto a LG agar plate to isolate a single colony. The isolated bacteria were identified by 16 S rDNA analysis by use of an identification service provided by Techno Suruga Lab (Shizuoka, Japan).

### Carbohydrate utilizing test

Carbohydrates utilizing tests for the thermophiles isolated in this study and *B*. *smithii* type culture were carried out by an Api 50CE kit (SYSMEX bioMérieux Co., Ltd., Tokyo) using a variety of carbohydrates indicated in Table 1. Bacterial cells cultivated in LB medium at 30°C for 2 days were washed with 0.85% (wt/vol) KCl and suspended in the medium provided in the kit at a final OD_660_ of 1.0. Two-hundred and fifty microliters of the suspension were used in the assay. Because LG and cellobiosan were not provided in the kit, these sugars were used at a concentration of 1% (wt/vol) in the separate microtubes.

### Bacterial treatment of RGM

The isolated bacteria were cultivated in the basal medium containing 5% (wt/vol) RGM as the sole carbon sources at 50°C for S-2701M and 60°C for S-1501M and S-65801. The culture media after several times were centrifuged to remove the cells. The supernatants were filtrated with a 0.2 μm filter and subjected to HPLC analysis. HPLC analysis was performed using an Ultron PS-80N column (Shimadzu GLC Ltd., Tokyo, Japan) at 80°C with deionized water as the mobile phase (0.3 ml/min). Saccharide fractions were detected by a refractive index detector.

### Enzyme assay

Bacterial cells grown in the LG medium were suspended in 0.1 M potassium phosphate buffer (pH 7.0) containing a protein Inhibitor cocktail (Complete, EDTA-free, Roche Diagnostics K.K., Tokyo, Japan). The cell suspension was disrupted with glass beads and centrifuged at 20,700 × *g* for 5 min, and the supernatant was used as crude extract. The reaction mixture for NAD-dependent LG dehydrogenase activity was composed of 100 mM Tris-HCl (pH 8.0), 2 mM β-NAD^+^, 0.6 M LG, and an appropriate amount of crude extract. LG kinase activity was measured by an enzyme coupling system in a reaction mixture containing 100 mM Tris-HCl (pH 8.0), 20 mM ATP, 100 mM MgCl_2_ · 7H_2_O, 2 mM β-NADP^+^, 2 unit/ml glucose-6-phosphate dehydrogenase (from yeast, Oriental Yeast Co., Ltd., Tokyo, Japan), 0.6 M LG, and an appropriate amount of crude extract. In both enzyme activities, the increasing absorbance at 340 nm in the reaction mixture was measured at 50°C for S-2701M and 60°C for S-1501M and S-65801. One unit of the enzyme activity was defined as the amount of the enzyme that catalyzed one mole of NADH or NADPH formation per min. Glucose-forming activity was determined by detecting glucose in the reaction mixture with a Glucose CII Test Wako (Wako Pure Chemical Industries, Ltd., Osaka, Japan).

### Compliance with Ethical Standards

This article does not contain any studies with human participants performed by any of the authors.
